# Solid-pseudopapillary tumour of the pancreas as a rare cause of gastric outlet obstruction: a case report

**DOI:** 10.1186/1757-1626-1-374

**Published:** 2008-12-04

**Authors:** Michael EC McFarlane, Joseph M Plummer, Jacqueline Patterson, Franz K Pencle

**Affiliations:** 1 Department of Surgery, Radiology, Anaesthetics and Intensive Care, University of the West Indies, Mona, Jamaica

## Abstract

The presence of a large bulky pancreatic tumour in a young female should raise suspicions of the diagnosis of solid-pseduopapillary tumour of the pancreas.

This rare tumour has the characteristics of a low-grade malignancy with indolent behaviour. Most patients present with vague non-specific abdominal pain resulting in delayed diagnosis. The light microscopic features show solid areas alternating with pseudopapillary formations. Metastases are frequently amenable to resection.

Favourable prognosis with long-term survival has been shown even in patients with metastatic disease. Herein we present the case of a 21 year-old female patient of Afro-Caribbean extract who presented with gastric outlet obstruction from a large pancreatic tumour.

## Background

Solid pseudopapillary tumors of the pancreas are rare and are often suspected in young females presenting with an abdominal mass. They are rarely found in children and commonly occur in women in the second to fourth decade of life. The pathogenesis is thought to result from cells of the endocrine pancreas though some investigators have postulated origin from the exocrine pancreas. These tumours have a long asymptomatic period and are usually detected when they have grown to a large size. Despite this presentation these tumours have low malignant potential. Metastases which are rarely seen occur mainly to the liver. It is therefore mandatory to establish a diagnosis and to attempt surgical excision even in large or metastasising tumours, since complete excision offers an excellent prognosis.

## Case report

A 21 year-old female of Afro-Caribbean extract presented with a 5-week history of intractable right- sided abdominal pain radiating to the back and associated with vomiting of bile-stained material. She gave a 1-week history of weight loss and jaundice. Her height was 165 cm and her weight 61 kg.

On examination she was mildly icteric and anaemic. The abdomen was flat and a tender immobile epigastric mass 5 cm × 7 cm was identified. The haemoglobin was 9.1 gm/dl and the partial thromboplastin time was elevated at 48.1/30.1.

The electrolytes, amylase and liver function tests were normal apart from elevation of the bilirubin with total bilirubin of 33 mmol/l and direct bilirubin of 12 mmol/l. Carcinoembryonic antigen (CEA) and CA 19-9 results were not available preoperatively.

**Figure 1:**
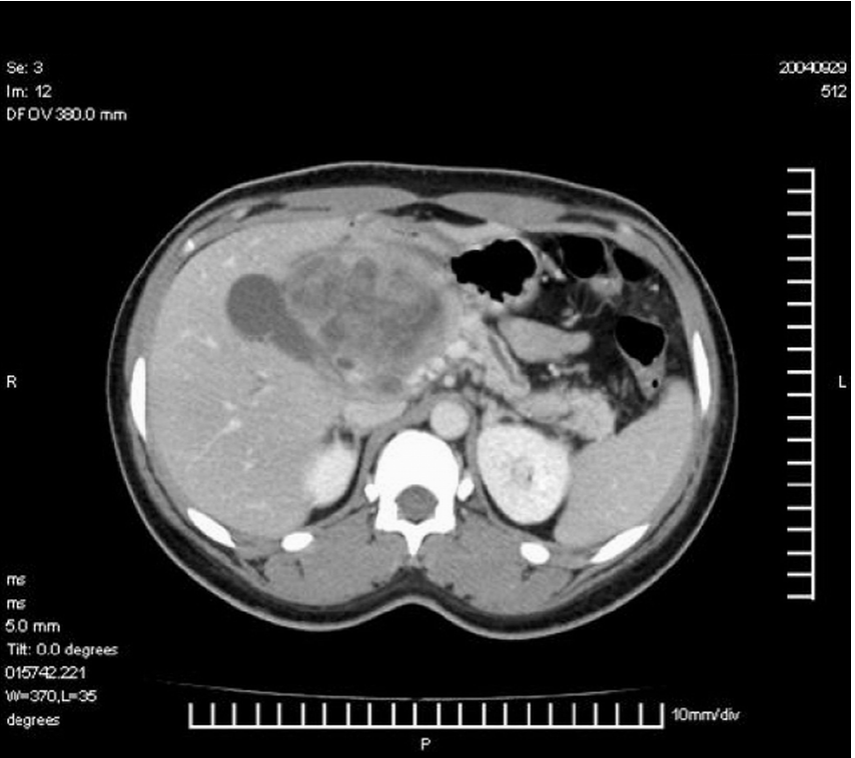
Contrast enhanced CT scan of the abdomen demonstrating a large inhomogenous pancreatic tumour.

**Figure 2:**
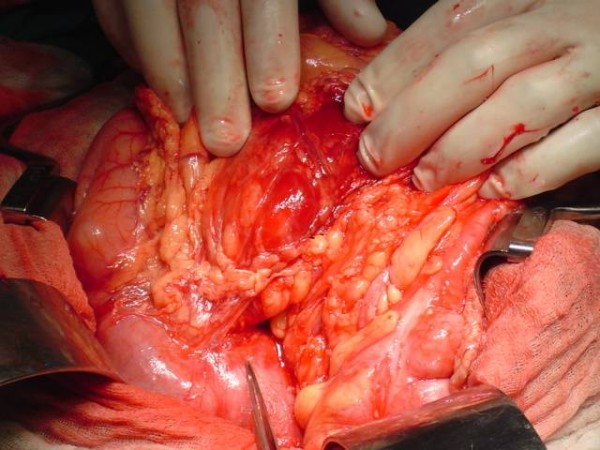
Operative picture showing a hard 8 cm diameter mass arising from the head of the pancreas displacing the duodenum anteriorly.

Abdominal ultrasound confirmed the presence of an abdominal mass in keeping with the physical findings. The CT abdomen identified an 8.1 × 7.7 × 7.5 cm mass in the head of the pancreas compressing the third part of the duodenum. [Figure 1] There were no other masses or lymph node involvement. A diagnosis of solid pseudopapillary tumour of the pancreas was suspected. Following preoperative preparation the abdomen was explored through a bilateral subcostal incision. There was a hard 8 cm diameter mass arising from the head of the pancreas displacing the duodenum anteriorly. [Figure 2] Hard mesenteric and celiac nodes were present and the gallbladder was markedly distended. There was complete encasement of the superior mesenteric artery by tumour. Frozen section analysis revealed a benign pancreatic tumour. Surgical debulking was considered but was not attempted due to marked encasement of the superior mesenteric vessels and obliteration of suitable planes of dissection. The tumour was deemed unresectable and a Roux-en-Y cholecysto-jejunostomy and gastroenterostomy performed.

Her postoperative course was uneventful. The histology confirmed the diagnosis of solid pseudopapillary tumour of the pancreas. Adjuvant chemotherapy and radiotherapy were not offered. The patient remained well after a three-year follow-up period.

## Discussion

Solid pseudopapillary tumours of the pancreas are rare. A total of 450 reports of solid pseudopapillary tumour of the pancreas have been reviewed in the literature since it was first described by Frantz in 1959.[1] The tumour has been identified by a number of synonyms including Frantz's tumour, solid and cystic acinar tumour, papillary epithelial neoplasm, and solid and papillary epithelial tumour or neoplasm. The tumour is thought to arise from ductal or acinar origin and has been mainly described in young women. Reports of the tumour in young children have also been made.[2] Most reports of these tumours occur in patients in the third decade of life with an age range of 12–79 years.[3,4]

The majority of patients present with vague abdominal symptoms, resulting in a delay in presentation and diagnosis. The tumour exhibits typical features on light-microscopy. The cystic appearance on gross examination may present a diagnositic dilemma with cystic neoplasms of the pancreas. Immunophenotyping has been used to differentiate these tumours from other pancreatic neoplasms. Solid-pseudopapillary tumours test positive for vimentin, neuron-specific enolase, α_1_-antitrypsin, and α_1_-antichymotrypsin and are negative for chromogranin, epithelial membrane antigen, and cytokeratin, insulin and glucagon.

There are no clear histological features, which establish the clinical behavior of these neoplasms. The tumours exhibit low-grade malignant potential and metastasize infrequently. Local recurrence has been reported in less than 5% of cases.[5] Mestastases typically occur in the liver, lymph nodes and peritoneum. Because these tumours rarely invade adjacent structures, even large tumours have been shown to be resectable. Rarely vascular invasion of the superior mesenteric artery or portal vein are encountered and may limit resectability. Complete resection of local disease is curative. Even patients with residual disease or metastases have been reported to have long-term survival following surgical treatment. Very few reports of the use of chemotherapy or radiotherapy for these tumours exist with only limited response.[6,7]

## Conclusion

The identification of a large bulky pancreatic tumour in a child or woman should raise suspicions of solid pseudopapillary tumour of the pancreas. Because of the indolent nature of these tumours and the low malignant potential, aggressive attempts at complete surgical resection are warranted. Large tumours are usually resectable and size does not predict outcome. Surgical bypass may be the only feasible option in patients with large tumours where the risks of attempting resection or debulking may be associated with overwhelming morbidity.

## Abbreviations

Cm: centimeters; kg: kilograms; gm/dl: grams/deciliter; mmol/l: millimoles/litre; CT: computed tomography.

## Consent

Written informed consent was obtained from the patient for publication of this case report and accompanying images. A copy of the written consent is available for review by the Editor-in-Chief of this journal.

## Competing interests

The authors declare that they have no competing interests.

## Authors' contributions

All contributors contributed in the authorship, review, and compilation of this case report. All authors read and approved the final manuscript. The authors are abbreviated as follows: MECM, JMP, FKP and JP.
